# RETRACTION: “Mechanical stretch promotes antioxidant responses and cardiomyogenic differentiation in P19 cells”

**DOI:** 10.1155/term/9851972

**Published:** 2025-08-01

**Authors:** Journal of Tissue Engineering and Regenerative Medicine

RETRACTION: J. Cheng, Q. Zou, Y. Xue, C. Sun, and D. Zhang, “Mechanical stretch promotes antioxidant responses and cardiomyogenic differentiation in P19 cells,” *Journal of Tissue Engineering and Regenerative Medicine*, 2021 (2021): term.3184, https://doi.org/10.1002/term.3184.

The above article, published online on 20 March 2021 in Wiley Online Library (https://wileyonlinelibrary.com), has been retracted following the agreement of the journal's Chief Editor Catherine Kuo; and John Wiley & Sons Ltd.

The retraction has been agreed following an investigation of the concerns raised by Hoya Camphorifolia on PubPeer [1], which identified concerns regarding Figures 4 and 5.

Specifically, similarity has been identified between the Western blot bands corresponding to SIRT 1 and GAPDH in Figure 4(b) to c-Casp 3 and c-Casp 9 bands in Figure 3(d) of [2]. Unexpected similarities were also identified between the cTNT and Cx43 bands in Figures 5(b), 5(d) and the Bcl-2 and Bad blots in Figure 3(d) of [2].

As a result of the investigation, the data and conclusions of this article are considered unreliable.

The authors were informed of the decision to retract the article but did not provide a response.
